# User Experiences With a Social Robot for Cardiometabolic Risk Assessment in a Community Setting in Uppsala, Sweden: Qualitative Semistructured Interview Study

**DOI:** 10.2196/87025

**Published:** 2026-10-06

**Authors:** Solveig Dolores Bruchhof, Helle Mölsted Alvesson, Jonas Idevall Hagren, Katie Winkle, Laran Matta, Marcus Göransson, Steve McKeever, Meena Daivadanam

**Affiliations:** 1 Department of Women’s and Children´s Health, Faculty of Medicine, Uppsala University, Dag Hammarskjölds väg 14B, Uppsala, Sweden, +46 72 999 97 20; 2 Department of Global Public Health, Karolinska Institutet, Stockholm, Sweden; 3 Department of Information Technology, Uppsala University, Uppsala, Sweden; 4 Department of Informatics and Media, Uppsala University, Uppsala, Sweden

**Keywords:** cardiometabolic disease, social robot, socioeconomically disadvantaged, health innovation, risk assessment, FINDRISC, type 2 diabetes, artificial intelligence

## Abstract

**Background:**

Cardiometabolic diseases (CMDs) are a major health concern worldwide, with people living in socioeconomically disadvantaged areas being disproportionately affected. Knowing one’s risk status for developing a CMD can help individuals in delaying or preventing the disease. Innovative tools and strategies such as the use of AI-based technology are needed to improve inclusivity, cost-effectiveness, and sustainability of screening processes.

**Objective:**

This study aimed to assess users’ perceptions of the usability and acceptance of the social robot “Furhat” for cardiometabolic risk assessment within a socioeconomically disadvantaged community in Uppsala, Sweden.

**Methods:**

In this qualitative study, 13 semistructured interviews were conducted with participants living in socially disadvantaged areas within Uppsala, after they completed a CMD risk assessment delivered by the social robot. The study was conducted in one of the socioeconomically disadvantaged areas in Uppsala from October 19 to 26, 2023. Participants were purposefully sampled at different community events via phone or on the street to achieve the desired variation regarding personal characteristics such as age, gender, or area of living. The used interview guide for this study consisted of questions regarding the demographics, participants’ experiences with the robot screening, and suggestions for improvements. A framework analysis approach was applied to the data.

**Results:**

Two themes were developed. The first theme *Self-versus-others: a matter of perception, norms, and assumptions* includes participants’ perceptions of who would use the robot and why, if it was installed in the community. Participants perceived older individuals, immigrants, and, in some instances, women to be less interested in the robot screening or as having a harder time participating in it. However, there were between-group variations, especially among men and women, with participants often discounting opinions ascribed to their group by others. The second theme *The robot is good, but humans are better* captures participants’ actual experiences of the interaction with the social robot once they have started the interaction. Although participants enjoyed the interaction with the robot, perceived it as nonjudgmental, and would recommend it to others, most would prefer conducting the screening with a human. Two major contributors for this preference were language barriers experienced during the robot-participant interaction, as well as their wish for more interactive, emotionally responsive, and communicative features of the robot.

**Conclusions:**

This study provides insights into users’ perceptions of the usability and acceptance of a social robot for risk assessment in a community context, which can help inform the further development of this technology for health screening in a community context. In the future, the robot could be further developed to conduct risk assessments in multiple languages and offer a broader service to users, such as providing appropriate information related to healthy and active living.

## Introduction

Social robots, which are typically powered by AI [1], are being increasingly used for health-related purposes, such as providing support for older adults who experience depression [2], or dementia [3], and for health screenings for mild cognitive impairment [4], or autism [5]. Using social robots for health screening has the potential to positively affect vulnerable and disadvantaged groups, who are often overlooked in research [6]. Despite this potential, most studies on social robots for health screening have been conducted in health care facilities or retirement homes rather than in community spaces [7]. Given the demonstrated effectiveness of community-based screening in reaching disadvantaged populations, further research is needed to examine user experiences with robot-facilitated screenings in these settings. Such deployments of social robots require extensive user testing for acceptance, correctness of content, and ethicality of use [8].

Users interact with health care technologies in diverse and complex ways, shaped by both their practical needs and emotional responses. According to research by Lupton [9], users often value health care technologies for their convenience, accessibility, and ability to offer personalized health insights, such as through self-tracking devices or digital health platforms. However, they also express concerns about trust, data privacy, and the depersonalization of care, as these tools may replace or reduce direct human interaction [9]. Despite these reservations, users primarily rely on health care technologies to monitor their health, access reliable medical information, and connect with support networks, reflecting a mix of empowerment and ambivalence in their engagement with these tools [10]. There are different frameworks aimed at explaining users’ interaction and acceptance of a certain technology [11]. One such framework is the Unified Theory of Acceptance and Use of Technology (UTAUT) framework, which aims to explain users’ behavioral intention of a technology as well as their actual use behavior [11].

Cardiometabolic diseases (CMDs), such as type 2 diabetes (T2D), stroke, and coronary heart disease, are among the most common and preventable conditions worldwide [12]. They share modifiable risk factors such as unhealthy diet, physical inactivity, smoking, hypertension, and obesity [13]; yet, they continue to account for high mortality and morbidity rates globally [12].

Screening for cardiovascular disease and T2D is cost-effective and facilitates early health behavior changes, potentially delaying disease onset and complications [14,15]. Up to 80% of CMDs, which often co-occur due to shared metabolic pathways, are preventable through lifestyle modification [16,17]. In Sweden, cardiovascular disease remains the leading cause of death, with approximately 200 deaths per 100,000 population in 2023 [18]. Prediabetes affects 12% of adults, with one-third being undiagnosed [19], while diabetes prevalence is 5.8%, predominantly T2D [20]. Key risk factors such as physical inactivity (47%) and adiposity (18%) [21] show marked social and gender disparities [22], disproportionately affecting foreign-born populations and disadvantaged suburbs [23,24]. Targeted strategies are needed to enhance screening access among these high-risk groups for timely CMD detection. Two primary screening approaches exist for early CMD detection: opportunistic screening, typically conducted in health care facilities, and community-based screening, which reaches individuals outside of formal health care settings [15]. In Sweden, a comparative study found that open community-based screening for T2D was better able to reach certain high-risk groups than facility-based opportunistic screening [25]. This included non-European immigrants with a higher genetic susceptibility to T2D and younger persons at high risk for T2D [25]. Additionally, type of screening tool (questionnaires with or without biochemical tests) and modality of screening (web-based, in-person, and self-administered) also influence who accesses screening opportunities and how much it would cost. However, little remains known about how to effectively implement equitable and accessible screening approaches in diverse community settings, particularly for underserved populations.

This study aims to critically examine how users from socioeconomically disadvantaged neighborhoods in Uppsala perceive their interactions with a social robot for CMD risk assessment and to assess the implications of these perceptions for the usability and acceptability of such technology in community-based health screenings.

## Methods

### Study Setting

Uppsala municipality includes 22 neighborhoods, with around 52,000 inhabitants, identified as socioeconomically disadvantaged [26]. These neighborhoods have higher unemployment rates, a higher proportion of residents receiving financial assistance, higher illness rates, lower household income, a higher proportion of economically vulnerable children and youth, lower percentage of residents with a postsecondary education [26], and a high proportion of non-European immigrants [27]. The robot screening was set up in one of these 22 neighborhoods, while the study participants came from 5 of the 22 neighborhoods. The robot screening was therefore conducted in the setting and population intended for its future implementation. To the best of the authors’ knowledge, this study is the first of its kind to explore users’ perception of the CMD risk assessment with a social robot in a community context.

### Study Design

This was an exploratory qualitative study where data were collected through semistructured interviews [28]. The SRQR (Standards for Reporting Qualitative Research) checklist is reported in Checklist 1. The study is guided by a pragmatist research paradigm. Pragmatism focuses on what works in practice and views knowledge as context-dependent and shaped by experience [29]. This fits the study well, as the goal was not to test a theory or discover universal phenomena but to understand how participants experienced their interaction with the robot in a specific context. The choice of framework analysis with mixed deductive-inductive coding reflects this stance: UTAUT was used as a practical tool to structure the analysis, while inductive coding allowed for new themes to emerge directly from the data [30].

The study was conducted as part of formative studies in the PREVENT project, which aims to reduce the risk for CMDs in high-risk populations within socioeconomically disadvantaged neighborhoods in the municipality of Uppsala through cocreating, implementing, and testing community-based support for healthy and active living [31].

The social robot used for this study was the “Furhat” robot, developed by a Stockholm-based company, consisting of a head (which can display different facial features and expressions) and a loudspeaker [32]. The expressions and movements thereby mimic human behavior. It can be programmed to have fluid conversations in different languages [32].

### Participant Recruitment and Inclusion Criteria

Participants were selected using purposive sampling with specific inclusion criteria to ensure relevance to the study’s aims. Through this variation, participants’ characteristics, experiences, and perceptions could be explored and compared between members of different groups. Eligible participants were aged between 30 and 75 years, lived in a selected area in Uppsala, and spoke Swedish (easy Swedish which is often spoken by people new in Sweden). Three participants also had a translator with them who helped them during the interaction and the interview. The age range of 30-75 years was chosen to reflect the demographic most at risk for CMDs, as individuals aged 30 years and older from regions such as the Middle East, Sub-Saharan Africa, and Southeast Asia have been shown to have a higher risk of developing CMDs [33]. The upper age limit of 75 years was set to reduce the likelihood of including individuals with neurodegenerative conditions, such as dementia or cognitive impairment, which could interfere with their ability to engage with self-reporting screening tools [34]. Easy Swedish was included as a requirement to minimize the complexity of introducing a social robot with speech capability to a multicultural and low-income suburb where many different languages are spoken.

### Data Collection

The screening and interviews were conducted over a 1-week period from October 19 to 26, 2023. Each participant was scheduled to conduct the screening and the interview during a specific time slot in this week. The risk assessment and interviews took place at the locale of a local civil society organization. For this study, the social robot “Furhat” was programmed to conduct the risk assessment using the standard Finnish Diabetes Risk Score (FINDRISC) questionnaire [35]. FINDRISC is a validated tool to assess people’s risk of developing T2D and, by extension, CMDs, within the next 10 years [36,37], due to common risk factors and causative mechanisms. It is an effective noninvasive tool used for screening in both health facilities and outside, such as community settings. FINDRISC includes a total of 8 questions that include demographic variables (age), anthropometric measures (BMI and waist circumference), behavioral measures (fruit and vegetable consumption and physical activity), and personal and family medical history (intake of antihypertensive drugs, high blood glucose, and family history of diabetes). Responses from each individual are scored, and a score of 13 or higher indicates a high risk for T2D [35] and is found to correlate closely with worsening values across various clinical and anthropometric parameters related to risk for T2D and CMDs [37]. The risk assessment with the robot was conducted in easy Swedish. Most interviews were conducted in Swedish or English, but 3 participants also had a friend or family member to help with translation to their mother tongue.

During the robot screening, only the male team member in charge of the technical aspects of the robot (“wizard”) and the participant remained in the test room. Participants were informed of this arrangement before providing consent. The test was set up in a way that the robot and the wizarding station were on opposite ends of the room to make participants focus on the robot instead of the wizard (Figure 1). During screening, the wizard tried to interfere as little as possible. However, some participants asked him when they did not understand something, in which case he responded. In one case, the robot failed to register voices, so the wizard entered the answers on his laptop manually. This was, however, not noticed by the participant as the robot was still able to ask questions. After the risk assessment, the interviewer returned to conduct the interview, while the wizard left. The interview was conducted next to the robot, so that participants could look at it during the interview to remember their feelings and experiences during the robot screening more easily (Figure 1). After the interview, the FINDRISC scores were explained to participants.

**Figure 1.**
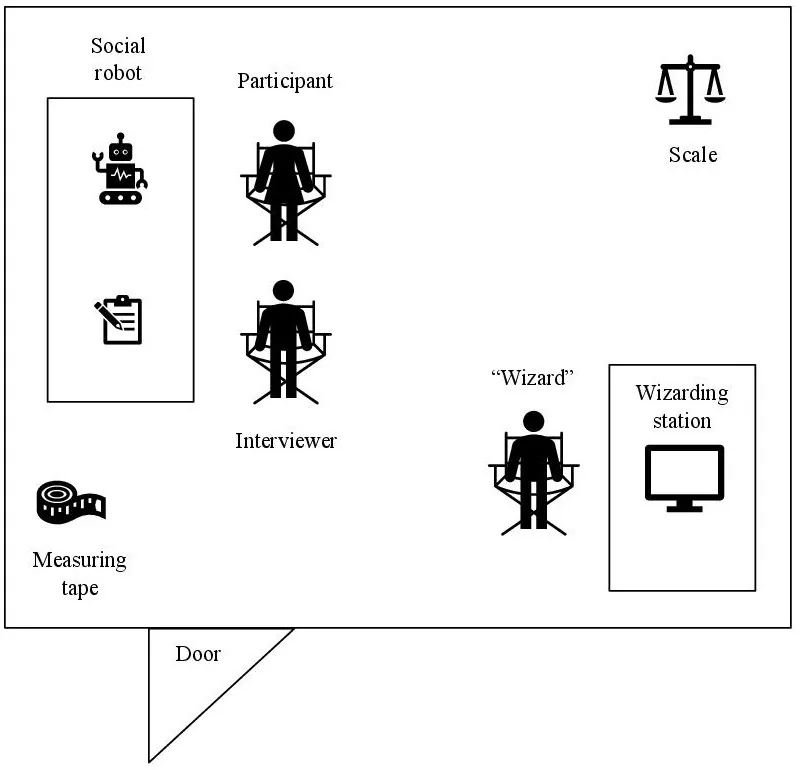
Setup of test and interview room.

The research process from obtaining consent to robot screening and the interview that followed took approximately 45 minutes per participant. Sample size was assessed using the concept of information power. Based on 4 of its 5 items (a narrow aim, the specificity of the experience studied, application of an established theory, and quality of dialogue based on strong and clear communication on a narrow topic), we assessed the 13 interviews to hold sufficient information power for the purpose of this study [38].

The interview guide used for this study included questions about participants’ demographics, their experiences with robot screening, suggestions for improvement, and their believed usefulness of robot screening in the community (Multimedia Appendix 1). The interviews were conducted by 1 member of the research team who was also responsible for fieldwork in the study area within the PREVENT project (JIH). All interviews were audio-recorded.

JIH is a Swedish-born, male anthropologist. At the time of the interviews, he had conducted 1 month of full-time participatory fieldwork in the study setting documented in field notes [39]. The fieldwork revolved around informal “deep hanging out” [40] in participants’ everyday lives. Details on the fieldwork and fieldwork activities have been published elsewhere [41]. With the exception of 2 participants recruited through other channels, JIH had met all participants more than once before the interviews and had informal interviews with them. This prior relationship may have facilitated rapport and openness during the interviews [42]. At the same time, the familiarity between JIH and participants may also have shaped both what participants chose to share and how their accounts were interpreted during the interview. Thus, while JIH contributed ethnographically grounded insight into the data, SDB, who led the analysis of the collected interview material, brought interpretive distance from the fieldwork and interview encounters. This division of roles supported a reflexive analytic process in which interpretation could be grounded in local contexts while also being questioned from a less embedded position.

### Data Analysis

The interviews were transcribed in Swedish and translated into English. Since some of the participants had a translator with them, 3 interviews had passages in Arabic, which were transcribed in Arabic and translated to English. All transcriptions and translations were performed by trained university students, native or fluent in the corresponding languages. Interviews were transcribed verbatim, with spot checks and back translations performed on the first transcripts (particularly those in Arabic to confirm the correct meanings). The interviewer later reviewed the translated transcripts to ensure that the translations retained the meaning and content of the original interviews. The framework analysis method was used to analyze the data in accordance with the UTAUT framework [30].

The UTAUT framework consists of 4 core determinants—performance expectancy, effort expectancy, social influences, and facilitating conditions (Figure 2)—that directly affect behavioral intention and use behavior. Behavioral intention describes the user’s intention to use the technology in the near future, while the actual use of the technology is called use behavior in the framework [11]. Performance expectancy is defined as the extent to which users believe that using the technology will help them achieve gains, in this case for their health [11]. Effort expectancy describes the user’s ease of interaction with the technology [11]. Social influences refer to how much the user believes that important others think they should use the technology [11]. Facilitating conditions refer to the user’s belief that there is technical or organizational infrastructure in place to support them in using the technology [11].

**Figure 2.**
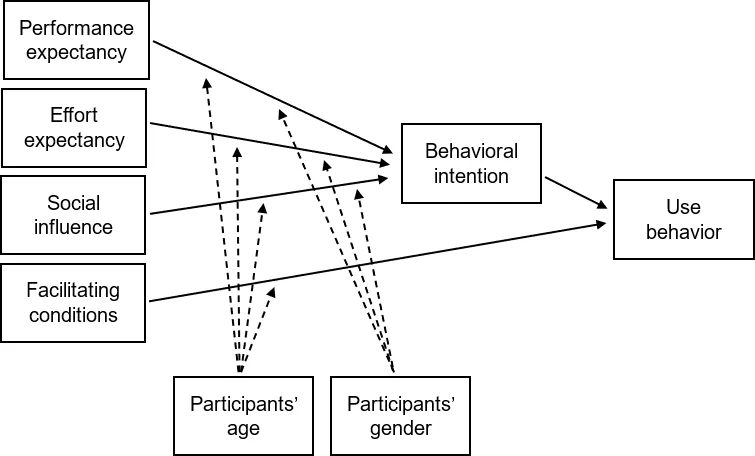
Modified Unified Theory of Acceptance and Use of Technology (UTAUT) framework reflecting study-specific constructs (voluntariness of usage and users’ experience are excluded due to irrelevance) (adapted from own presentation based on UTAUT framework by Venkatesh et al [11]).

For the coding, a mix of deductive and inductive approaches was used. An initial codebook was created by SDB, using a deductive approach guided by the UTAUT framework and informed by the interview guide and recurring topics identified during initial familiarization with the data [30]. The 4 UTAUT determinants served as the basis for the initial codebook. This codebook was then used by JIH, MG, and SDB to independently code 1 transcript. After discussing differences and similarities in the coding, the codebook was revised by SDB. Where participant responses did not fit within these predefined categories, new codes were added inductively, allowing themes to emerge directly from the data. The coding was conducted across 4 rounds with the help of the open-source software “QualCoder (version 3.4)” [43], after which the initial UTAUT-based themes were recategorized due to overlaps, resulting in a revised set of themes that better reflected the data. The codebook can be found in Multimedia Appendix 2.

### Trustworthiness

To ensure trustworthiness, strategies addressing credibility, dependability, confirmability, and transferability were applied. Credibility was supported through investigator triangulation: 1 transcript was independently coded by 3 researchers (SDB, JIH, and MG) using an initial codebook, after which discrepancies were discussed and reconciled. This helped identify individual interpretive biases before coding the remaining data. Dependability and confirmability are accounted for by keeping an audit trail of the whole coding process, changes made in the codebook, and in the categorization of codes, as well as field notes from the interviewer and the wizard. Field notes and personal observations were used to continuously reflect on assumptions and minimize bias during the writing process. Transferability of the results was supported through a thick description of the sampling strategy and the research context [44].

### Ethical Considerations

The study received ethical approval from the Swedish ethical review authority (RN2022-04034-01). All participants provided informed written consent and were compensated with vouchers from supermarkets in the neighborhoods. Participants were informed about how their personal data were collected and stored. Interviews were audio-recorded and the data were anonymized. All data were stored on a secure Uppsala University server to which only the research team had access. Every person who transcribed, translated, or worked with the transcripts signed a confidentiality agreement.

## Results

### Participants

A total of 13 people participated in the study. All 13 participants were born outside of Sweden and had Swedish as their second language. The participants were born in Syria, Iran, Saudi Arabia, Sudan, Somalia, Eritrea, Yemen, and Kurdistan. Participant characteristics are shown in Table 1.

**Table 1.** Characteristics of participants.

Participant number	Age (years)	Sex	Living situation	Languages spoken
P1	59	Male	With family	Arabic, English, and Swedish
P2	54	Male	With family	Arabic, English, Tigrinya, Mariska, and Swedish
P3	43	Male	With roommate	Persian, English, Spanish, and Swedish
P4	52	Male	Alone	French, Arabic, and Swedish
P5	47	Female	With family	Arabic and Swedish
P6	33	Female	With family	Arabic, Swedish, and English
P7	51	Female	With family	Somali, English, and Swedish
P8	36	Female	Alone	Arabic, English, and Swedish
P9	52	Female	Alone	Arabic, Tigrinya, Saho, and Swedish
P10	65	Male	With family	Kurdish, Arabic, and Swedish
P11	50	Female	With family	Arabic and Swedish
P12	35	Female	With family	Arabic, Swedish, English, and Turkish
P13	47	Female	With family	Kurdish, Arabic, sign language, and Swedish

### Findings

#### Findings Overview

The results consist of 2 themes and 8 subthemes, illustrated in Figure 3. The first theme represents the steps that would allow or hinder a community member from approaching the robot if placed in the community (outer circle in Figure 3). The second theme captures participants’ actual experiences with the social robot once they have started the interaction (the inner circle in Figure 3).

**Figure 3.**
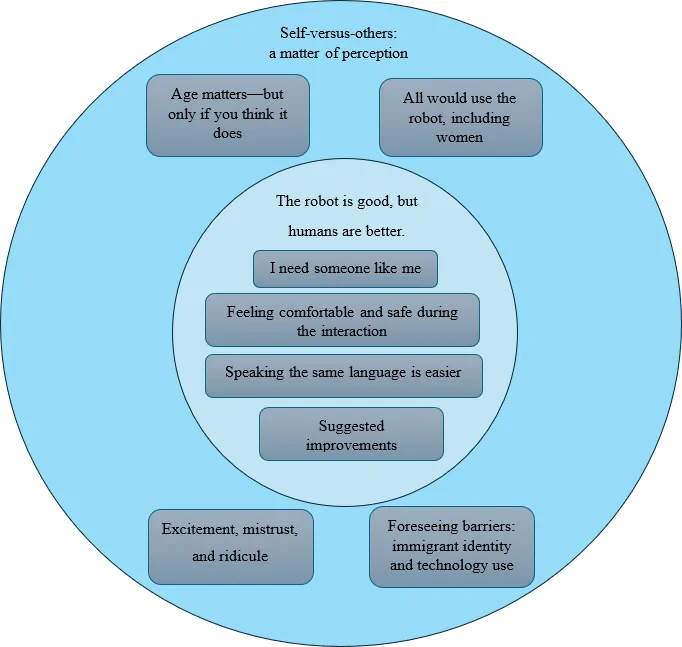
Visual representation of the results of the framework analysis. The outer circle encompasses the factors that influence community members’ willingness to approach the social robot for the health screening. The inner circle includes the findings related to the community members’ actual interaction with the robot.

#### Self-Versus-Others: A Matter of Perception, Norms, and Assumptions

The first theme explores how social norms and personal assumptions shaped participants’ engagement with the robot for health screening—both in terms of their own willingness to interact with it and their expectations of how others in their community would react to it. This aligns with the UTAUT outcome of behavioral intention, which in the context of this theme was particularly shaped by social influence and performance expectancy. The beliefs about who would use the robot screening were influenced by their ideas about age, gender roles, and social identity, and their assumptions of others’ skills and affinity in interacting with the robot. The latter aspects—particularly social identity and projected assumptions about others’ preferences—emerged inductively and extended beyond the core constructs of UTAUT.

##### Age Matters—but Only if You Think It Does

Age was often mentioned as a factor influencing the acceptance and ease of interaction with the robot for risk assessment. Young adults were, in general, considered to be most interested in robot screening, while older adults were believed to prefer human interaction. Younger people were seen as being more familiar with using technology and having greater trust in it. Older age was in general considered a barrier to the use of new technology, although a person’s mindset was described as being able to overcome that challenge. How participants viewed the effects of age on the use of technology therefore seemed to vary, depending on their own age and their perception of their age. One participant, for example, described people older than 60 years as old, while excluding himself (aged 59 years) from that group.

Age was perceived to influence not only others’ affinity and ease of interaction with the robot but also one’s own. Some of the older participants expressed that they perceive their age to negatively influence their ease of interaction with the robot, thereby supporting participants’ beliefs about age being a decisive factor in who would approach the social robot. However, some older participants also reported that they liked the robot screening, therefore supporting the importance of one’s mindset over one’s age.

*And there are some people who are even a little older, they read about these robots, and they want to see them physically. [...] It depends on how you take it. It depends on you. If you say no this is difficult, then it will remain difficult. But if you say no, it’s ok you can do it, then you can.*

[Participant 2, aged 54 years, male]

##### All Would Use the Robot, Including Women

Gender was another factor that was perceived to influence people’s affinity toward the use of a social robot for health screening in the community. Most participants believed that both men and women would be equally interested in the robot screening if it was installed in the community. However, 2 male participants had opposing opinions about women’s preferences. One of them, who showed an interest in risk assessment himself, believed that women would use the robot more because they cared more about their health. The other, however, assumed that women in general and older women, in particular, would use the robot less, since they would prefer humans who could show emotions. The latter participant seemed to view robots as lacking in the emotional depth that he believed women desired in interactions. The assumption that women would not use the robot was, however, refuted by female participants, who expressed their excitement over it.

*I think men would use the robot more. Women are emotional, are sensitive. They want doctors. They say: “I don’t do anything with robots, no!”. They think like that, I think. They think things are going very well with a human.*

[Participant 1, aged 59 years, male]

*All the people would use the robot. All the women too, including me. I think it is fantastic.*

[Participant 7, aged 51 years, female]

##### Foreseeing Barriers: Immigrant Identity and Technology Use

Although all participants were born outside of Sweden, they often excluded themselves when talking about the preferences of immigrants. The term “immigrants” seemed to have been used by participants to refer to those who could not speak fluent Swedish or those experiencing immigration issues. Some participants assumed that immigrants would prefer human interaction for the risk assessment. This general assumption of immigrants being uninterested in the robot screening was not reflected by the accounts of the participants themselves who, despite identifying as immigrants, demonstrated interest and enthusiasm. However, it is worth noting that participants who self-selected to take part may not represent the broader immigrant population, including those who might indeed prefer human interaction. One factor influencing this assumption was the language setting (easy Swedish) of the robot, and the second was the perceived need among immigrants for integrating with the host population through human interaction. Swedish was the second language of all participants, and they sometimes had difficulties understanding or answering questions during the screening. Some participants explained that learning Swedish was more difficult with age, making the interaction with the robot especially difficult for older immigrants.

*Many elders they don’t know Swedish in this area. They know a few words but if you think from my perspective [having difficulties understanding what the robot said myself], then maybe you could imagine that someone else would feel the same.*

[Participant 13, aged 47 years, female]

Additionally, some participants perceived immigrants as preferring human interaction partly due to a desire to integrate into Swedish society through human contact, thus also allowing them opportunities to practice their Swedish. Nonimmigrants, on the other hand, were assumed to not have similar integration needs as they were thought to already have good interactions with others through their schools or jobs. They were therefore assumed to prefer robot screening, which was seen as something new and interesting that they could take the opportunity to experience. One participant explained that immigrants might feel like they were not allowed to use the robot, suggesting that they may feel excluded from using the technology due to their social identity, perceiving it as not meant for them or irrelevant to their experience. Moreover, he believed that risk assessment would not be a priority for immigrants since they might have more pressing issues related to immigration or their families. These views reflect the participants’ own perceptions and assumptions about their communities, rather than verified preferences of these groups.

##### Excitement, Mistrust, and Ridicule

Furhat seemed to elicit a spectrum of varied reactions from the participants. None of the participants had any prior interaction with robots, but most described the experience as fun and expressed a positive attitude toward the robot, feeling excited and happy.

*It was fantastic. I’m 51 years old and this was my first time meeting a robot. I feel lucky. In my home country I have never met a robot. It was my first time here in Sweden.*

[Participant 7, aged 51 years, female]

Based on the participants’ positive experience and attitude toward robot screening, they believe that other community members would appreciate it, and they would recommend the robot screening to others in their community. However, some participants believed that there would be people who would not use the robot because they mistrust new technologies or were afraid of interacting with them. Some also voiced concerns about others trying to damage the robot if it was installed in the community. Some also expressed fear of being ridiculed for using the robot for risk assessment by other community members, suggesting that how they were perceived by others was an important factor influencing their decision to use the robot. They worried that their behavior would be negatively judged, which could discourage them from adopting this unfamiliar technology.

*Others will come and look at you, laugh at you “Ahahah he’s talking to the robot.” You don’t know what others think of.*

[Participant 12, aged 35 years, female]

##### The Robot Is Good, but Humans Are Better

This second theme describes participants’ experience of robot interaction, reflecting findings related to the UTAUT outcome of use behavior. It encompasses participants’ perceived differences between conducting the screening with a human versus a robot and experiences with language barriers during the interaction, both reflecting the constructs of effort expectancy and performance expectancy. The theme further extends beyond these core UTAUT constructs, incorporating participants’ feelings of safety and trust—which emerged inductively—as well as their suggestions for future improvements to the robot’s community installation, the latter reflecting the UTAUT construct of facilitating conditions.

##### I Need Someone Like Me

When asked to compare screening by a robot versus a human, the robot was perceived to have certain advantages, as it could work faster and more precisely. Because of its human-like face and expressions, it was experienced by some as looking and acting like a human. The interaction with the robot was described as similar to talking to health care professionals on the phone or in-person. Some participants also described the risk assessment as equally good with the robot as it would have been with a human, since they would both have asked the same questions. However, in spite of the advantages of robot screening described by the participants, most of them reported a preference for doing the risk assessment with a human. Both men and women emphasized that, unlike humans, the robot was unable to show emotions.

*I think there are big differences between robots and humans. There are emotions, many feelings when we talk to a person. But the robot has no feelings, only data. When there is eye contact you sympathize with me. You show me emotions, but the robot has no feelings. This is something bad, in my opinion. Maybe robots can be developed to do better than humans in the future, better than doctors? But we still need doctors, especially for feelings.*

[Participant 1, aged 59 years, male]

Participants considered that communication with humans would be easier, since they could ask them to speak slower or louder, translate questions, or read a person’s lips. The robot could only ask the programmed questions and could not respond to questions from users, which was perceived as a shortcoming. Some participants also expressed feeling more comfortable talking to humans because interacting with a robot was something new and unfamiliar, which stressed them. They could also clearly see and feel that the robot was just a machine, which was not described as positive.

*You can feel that there is no blood, the robot is just metal and plastic. The robot is good but contact with humans is better.*

[Participant 10, aged 65 years, male]

Some participants also described being uncertain about how to interact with the robot, which might have negatively influenced users’ ease of interacting with it. One participant did not know at first that the robot could not answer questions freely. Another reported that she did not know where to look when talking to the robot. She likely felt confused about whether to look at the robot’s eyes or the camera that the robot used to track users’ position.

##### Feeling Comfortable and Safe During the Interaction

Although 2 participants expressed slight mistrust in the robot, most described trusting it primarily in terms of its interactional qualities, confidentiality, and perceived functionality. Participants perceived the robot as nonjudgmental and unbiased during the screening, and several noted that they trusted it to keep their answers to sensitive questions confidential. Trust was also linked to beliefs about the robot’s effectiveness in providing reliable results. The skepticism expressed by some participants stemmed from broader doubts about other technologies, such as self-driving cars, and concerns about whether the robot’s results could be trusted. In general, more female participants reported trusting the robot and described feeling safe and comfortable during the interaction.

*I feel comfortable when talking with this robot, because I know that if he was like a human, he would judge me before I start talking. I am usually afraid of talking to people in a language I don’t know. But with him I felt comfortable.*

[Participant 9, aged 52 years, female]

Another factor influencing how comfortable participants felt during the interaction was the appearance of the robot. Although most participants, men and women alike, expressed that they liked the white-blue titan face and male voice displayed by the robot, some of the female participants expressed that they would prefer a female-looking and sounding robot, as this would make them feel more comfortable. The size of the robot elicited mixed reactions. While one participant wished for a bigger robot with a full body, another participant expressed that the smaller size made it less intimidating. The tone of voice and attitude of the robot were also described as enhancing trust and a feeling of comfort during the interaction. The robot was described as calm, friendly, polite, kind, smart, and professional. While the overall experience was positive, some participants reported feeling uncomfortable answering some of the more sensitive questions on, for example, weight in front of the wizard (who was present in the room during the interaction).

Most participants believed that the risk assessment with the robot was beneficial for themselves and others. Knowing one’s risk for CMDs was perceived as important and helpful.

*Maybe many need to know the risks of getting diabetes or other illnesses, so it’s easier to go to the health center or to some other place and ask. When talking to the robot and getting answers you become more confident or more comfortable that you’re doing the right thing in your life.*

[Participant 12, aged 35 years, female]

Being able to do the risk assessment in the community was also perceived as beneficial, as they could save time and money, compared with doing the screening in a health care facility.

*I think actually this [screening in the community with the robot] is more comfortable. I mean, if it’s located in some place, and you can just go inside without taking reception, anytime. And you can also trust the result–I think it’s very good. I mean, other than going a long way to be interviewed with a person, the robot screening can be very helpful.*

[Participant 3, aged 43 years, male]

##### Speaking the Same Language Is Easier

The risk assessment was conducted in Swedish, which was the second language of all participants. The language was perceived as a major factor influencing participants’ ease of interacting with the robot. Most participants reported at least some difficulties in understanding the questions asked by the robot, as well as the FINDRISC score given at the end. Participants often blamed themselves for not being able to understand the robot.

*The language of the robot was not poor, but my language is perhaps. I don’t understand, but it [the screening] goes very well with the robot, but the fault is with me, because I don’t understand.*

[Participant 1, aged 59 years, male]

Screening with a robot was, however, seen as helpful to ensure privacy, since users who were not fluent in Swedish could still do the assessment without a translator, which was seen as more accessible and attractive for immigrants. Aside from the barriers, participants also named several factors which they perceived as helpful during the interaction. The pronunciation of the robot was perceived as clear and easy to understand. They generally perceived the language recognition ability of the robot as good, and the option to ask it to repeat a question was also perceived as helpful.

##### Suggested Improvements

Participants had several suggestions to improve the use of social robots in risk assessment. Participants suggested that the robot could have multiple language competencies and that users could choose the language for the screening, since the language barrier was felt to be a major concern. The most mentioned languages were Arabic and English. It was also suggested that users could choose the speed of the conversation and have the robot talk slower when needed.

*I think the robot would be easier to use if it could speak multiple languages. Today, for me, for example, I’m new, right? I still need to practice my Swedish. So, Arabic would be good, if it’s possible, but English more or less like an international language. I did not understand the result, but if it would have been in English, I could have caught it.*

[Participant 2, aged 54 years, male]

While robot screening allowed for a feeling of safety and comfort, it was suggested that the robot be placed in a small room, without any staff inside, to ensure privacy during the screening process. It was also suggested to let the user choose the appearance of the robot, as some women expressed a preference to talk to a female version.

For this study, it was decided to have the robot give the FINDRISC score to the participants as a number, without an explanation about what risk status it translates to. This score was then explained to participants after the interview to ensure that participants fully understood their risk status but were not stressed about a possible high-risk status during the interview. Participants recommended that the robot give users a risk status instead of a score to further reduce the differences between robot and human screening. Expanding the screening beyond T2D or CMD risk assessment to cover other diseases was also suggested. This included recommendations to provide users with health tips and advice after completion of the risk assessment.

## Discussion

### Principal Results

Participants generally stated that they perceived the robot screening as positive but nevertheless most would have preferred doing the risk assessment with a human instead. Two major contributors for this preference were the language barrier between participants and the robot, and participants’ perception of the robot to be lacking in comparison with a human because of its limited programmed abilities, lack of emotions, and lack of responsive adjustment to people’s needs. However, participants generally perceived the robot as nonjudgmental, trustworthy, and keeping users’ information confidential, which had a positive effect on participants and made them feel more comfortable interacting with the robot for the risk assessment. Certain groups of people such as women, older adults, or immigrants were often perceived to be more reluctant or having a harder time using the robot, which was mostly not supported by the accounts of the respective groups.

### Comparison With Prior Work

Most studies investigating users’ interaction and perception of social robots or socially assistive robots are conducted with older adults living in retirement homes. The use of social robots in preventive and community-based health care settings has had very little attention so far. However, similar to this study, other studies found that users generally had a positive attitude toward using social robots but perceived them to have limited capabilities in interaction compared with humans [45,46]. Users especially criticized the robots’ inability to show emotions, display different facial expressions, respond to the users’ mood [46], or having only a limited amount of interactive functions [45], which is in line with the findings from this study. A study by Wu et al [47] in a gerontology ward in France found that participants had a negative attitude toward the socially assistive robot that was tested. They reported feeling uneasy with the technology, feeling stigmatized by others for using it, and did not perceive it as useful [47]. Although most participants in the PREVENT study reported a preference to interact with humans, they generally expressed a more positive attitude toward the robot. While some of the users of this study reported that they were afraid of others ridiculing them for using the robot or they were slightly distrusting of the robot, the majority expressed the opposite and stressed potential benefits of the robot screening in the community.

The findings of this study about the robot being perceived as friendly, trustworthy, nonjudgmental, and maintaining confidentiality are in line with findings from other studies where a social robot was tested in a retirement home [48] or as a language teacher [49]. A study by van Maris et al [50] found that users’ trust in robots increases over time. Some of the participants of the PREVENT study reported feeling slightly stressed since interacting with a robot was something new and unusual for them. Therefore, it is possible that repeated interactions with the social robot for health screening could enhance users’ trust and acceptance of it over time.

While most participants perceived women and men to be equally interested in the robot screening, women were sometimes perceived by men as preferring human interaction. The women themselves, however, refuted this view of them as being less interested in using a social robot for health screening. What is interesting here is not so much a gender difference in acceptance but rather the assumptions about emotions as motivators for seeking or avoiding risk screening with a robot. These assumptions reflect broader gendered stereotypes about comfort, relationality, and technology use. Existing literature shows mixed findings on gender preferences in social robot acceptance, with some studies reporting higher acceptance among women [51] and some among men [52,53]. Such differences may partly relate to the appearance and abilities of the robots tested, but our findings suggest that perceptions of emotional motivation may be equally influential in shaping expectations about who would use such technologies.

When it comes to age, participants in this study believed older individuals to be more hesitant toward interacting with the robot, whereas younger individuals were believed to be more interested and more competent with technology. While some of the older participants reported their age to be a hindering factor in the interaction, others believed that it was more a matter of one’s own mindset and interests. The age stereotype is also contradicted by a study by Kuo et al [53], which investigated attitudes of users toward a health care robot in New Zealand. Older people expressed similar positive attitudes toward using the robot as middle-aged participants, although older participants were less experienced with technology [53]. A study by Van Dijk [54] discovered that the motivation to use and acceptance of technology by older adults increases if the device is perceived to be useful and convenient. Therefore, the generally positive attitude of older people toward the social robot used in this study might be influenced by their belief about its usefulness and the ease of interaction with technology.

Another group that was seen as more reluctant to use the social robot for risk assessment if it was installed in the community were immigrants. However, the participants refuted this view themselves with their enthusiasm for and interest in the risk assessment with the robot. It is important to note here that all participants were volunteers, which means they were likely among the most open and technology-interested members of their community. Therefore, they may not represent those who would indeed prefer human interaction. Nevertheless, the contrast between participants’ assumptions about others and their own personal experiences suggests that stereotypes and societal norms may shape expectations more than actual user preferences. A systematic literature review on technology use among immigrants found a limited use of technologies in older immigrants compared with younger ones [55]. Older immigrants were found to be less interested in learning how to use certain technologies, as well as having more difficulties in using them [55], which is in line with the beliefs of the participants of this study about older immigrants. An aspect of the assumed disinterest among immigrants for robot-assisted risk screening was in how they included themselves or not in their own definitions or descriptions of immigrants. Participants often excluded themselves from the groups of immigrants when talking about preferences—defining “immigrants” as those who struggle with language or cultural integration. This aligns with Moffitt and Juang’s [56] research, whose participants described immigrants as people who “don’t speak the language well” or “stick only to their own cultural norms”—criteria they used to exclude themselves from the label.

The UTAUT framework, developed to explain users’ intentions to adopt a technology and their subsequent usage behavior [11], was valuable for structuring the deductive part of our analysis. It provided a useful lens to interpret how factors such as performance expectancy, effort expectancy, and social influence shaped participants’ experiences with the robot and ultimately shaped their behavioral intention and use behavior [11]. With regard to performance expectancy, participants, independent of age or gender, generally believed that using the social robot for the risk assessment will bring them benefits for their health. However, this positive perception was mostly associated with the community screening itself and not the robot screening per se. As for the effort expectancy, the language barrier was reported as the main barrier in the ease of interaction with the robot. The perception that it would be easier to communicate with a human might have contributed to participants’ preference of conducting the screening with a human. The language barrier was not dependent on participants’ gender, although older age was attributed with having more difficulties in learning Swedish. When it comes to social influences, some participants reported being scared that others would ridicule them for interacting with the robot, which could potentially make them more reluctant to use it. However, most participants felt safe and comfortable during the interaction with the robot. These feelings of safety and trust emerged as an important inductive finding, as they are not included in the UTAUT core constructs. These experiences did not differ by age or gender of participants. With regard to facilitating conditions, participants’ suggestions for improvements to the future installation of the robot—such as enhanced language support and more interactive and emotionally responsive features—highlight the infrastructural and contextual factors perceived as necessary for successful deployment. These findings illustrate how UTAUT can capture important aspects of acceptance in this setting. However, the UTAUT framework was initially developed to model behavioral intention and sustained technology adoption in contexts of repeated use, which is not applicable to a periodic activity such as cardiometabolic risk screening. The findings should therefore be understood as reflecting initial impressions rather than settled adoption attitudes, particularly for constructs such as performance expectancy and social influence, which are likely to be shaped by repeated exposure and experience over time. Participants’ beliefs about the robot’s benefits for their health or about how others in their community would react to it may shift considerably following multiple encounters, and a longitudinal study may therefore yield different findings. Furthermore, the framework did not fully explain participants’ broader beliefs about who in their community would (or would not) be willing to use the robot, which suggests that additional perspectives are needed to fully understand social meanings around robot-mediated health screening.

### Limitations

This study had several limitations, the main one being that the risk assessment with the social robot was conducted in Swedish, which was not the mother tongue of the participants. However, this being the first robot deployment in the community, use of Swedish as the operational language was a deliberate choice to reduce the complexity of interactions and the scope of the study. Since it was the first field deployment, we kept the interaction elements simple, with no added interpretative or explanatory elements where advanced language skills would have been necessary for both the robot and the participants. For this reason, the robot communicated the FINDRISC result as a numerical score only, without categorizing it into a corresponding risk level (eg, low, moderate, and high). While this simplified the interaction, it may have limited participants’ understanding of their diabetes risk, as the clinical significance of a given score is not necessarily self-evident without an accompanying risk category. The language bias was minimized by having a translator for the interviews, where possible. Consequently, the language barrier was a foreseeable outcome of the study design rather than an unexpected finding. Furthermore, it remains unclear whether the language difficulties reported by participants were specific to interacting with a robot in Swedish, or whether they would apply equally to any Swedish-language health encounter. Future studies deploying multilingual robot capability would help disentangle these 2 aspects.

Second, the wizard was present in the room and visible to the participants during the screening, although he placed himself outside their direct line of vision. Although he tried to interfere as little as possible, some participants instinctively turned to the wizard when they did not understand a question, which might have influenced participants’ ease of interaction with the robot. Additionally, some participants reported discomfort answering sensitive health-related questions in front of the wizard, which may have affected their responses. Furthermore, the wizard’s presence may have had broader consequences for the validity of the findings. Knowing that a human was available as a fallback could have inflated participants’ feeling of comfort and trust, as the interaction did not reflect a fully autonomous robot deployment. This reflects the early-stage nature of this deployment, where human oversight remains necessary to ensure technical reliability. Findings on acceptance and trust should therefore be understood as reflecting robot screening in a supervised context.

A further limitation concerns the conditions under which the interviews were conducted. Participants were interviewed immediately after the screening, in the same room as the robot, and by a researcher involved in the PREVENT project. These conditions may have encouraged socially desirable responses, as participants might have felt inclined to respond positively out of politeness toward the interviewer or reluctance to criticize the technology in its presence. This could partly explain the pattern observed in the findings, where participants frequently described the robot very positively while simultaneously expressing a preference for human interaction. Future studies could address this by introducing a time delay between the screening and the interview.

Furthermore, selection bias needs to be considered when interpreting the findings. Since all participants were volunteers, they were likely among the most willing and open members of their communities. This is particularly relevant for theme 1, where participants reflected on whether others in their communities would use the robot. Interestingly, some participants distanced themselves from the groups they were describing; for example, participants who were themselves immigrants often excluded themselves when talking about the assumed preferences of immigrants. While this reflects a nuanced and self-aware perspective, it also highlights that the views of those who might genuinely hesitate are not captured in this sample. The perspectives of those who declined to participate or would never have volunteered remain unknown, and claims about community-wide acceptance should therefore be interpreted with caution.

Moreover, as UTAUT was developed to model sustained technology adoption over time, findings based on a single postinteraction interview reflect initial impressions rather than considered adoption attitudes. Longitudinal follow-up studies examining whether initial perceptions translate into actual acceptance of social robots for health screening over time would strengthen the evidence base.

### Conclusions

This study provides insights into users’ perceptions of the usability and acceptance of a social robot for risk assessment deployed in a community context. Although participants enjoyed the interaction with the robot, perceived it as nonjudgmental, and would recommend it to others in their community, most would prefer conducting the screening with a human. Both the language barrier experienced during the robot-participant interaction and their wish for more interactive, emotionally responsive, and communicative features of the robot contributed to this preference. However, this needs to be considered in the context of developing and implementing cost-effective processes for at-scale population-based screening, which would most likely necessitate the minimization of human personnel.

## Supplementary material

10.2196/87025 Multimedia Appendix 1 Interview guide.

10.2196/87025 Multimedia Appendix 2 Codebook.

10.2196/87025 Multimedia Appendix 3 Prompts and responses of generative AI.

10.2196/87025 Checklist 1 SRQR checklist.

## Abbreviations

CMD: cardiometabolic disease
FINDRISC: Finnish Diabetes Risk Score
SRQR: Standards for Reporting Qualitative Research
T2D: type 2 diabetes
UTAUT: Unified Theory of Acceptance and Use of Technology
