# Supplementary material for: User Experiences With a Social Robot for Cardiometabolic Risk Assessment in a Community Setting in Uppsala, Sweden: Qualitative Semistructured Interview Study
Source: J Med Internet Res. 2026 Oct 6;28:e87025. doi: 10.2196/87025 (PMC13641321; doi:10.2196/87025)
Supplement: Supplementary file 1 [file jmir-v28-e87025-s001.pdf]

Multimedia Appendix 1 - Interview guide – Social Robotics

2023-10-23

Sociodemographic questions

1. How old are you?
2. What is your gender?
3. Do you live alone or with family?
4. Were you born in Sweden? If not, when did you move to Sweden and where did you move from?
5. What languages do you speak?
6. In what area of Uppsala do you live?

Semi-structured Interview guide

What you just did with the robot was a screening for type 2 diabetes. Screening involves estimating the risk of developing a disease. Based on the interaction (conversation) you just had with the robot, we want to ask you some questions.

1. What did you think of this screening experience with the robot?
    a. How would you describe the robot?
        i. Friendly, competent, stressed?
    b. How could this robotic screening process be improved?
        i. Language? How well did it understand you?

There are many different types of screening that you can do. What you just did was a screening for type 2 diabetes. Now I would like to ask you if you have done any screening before and how you think it differs when you are screened by a robot instead of a human.

2. Have you done any screening in the past, but with a human being?
    a. If yes, how is it different to be screened by a human instead of a robot?
    b. If no: would you have preferred to be screened by a human instead of a robot?
        i. Why X?
        ii. When X, when Y?

3. At the end of the conversation with the robot, did you receive a screening result? Did you understand what your result was?
    a. How did it feel to get the result from a robot?
        i. Do you think it would have felt different to get it from a human?

        ii. How? Why?

4. Based on your experience with the robot, would you recommend this robotic screening for diabetes to your friends or relatives?
        i. Why/why not? Which relatives? All friends?
    b. If we were to exhibit this robot in area 1 (name of the area was anonymized for this publication) or a similar place in Uppsala, what do you think people would think of it?
        i. Do you think there are any people with whom this robot screening would work better or worse?
            1. Are there any people who would like it less or more?
            2. Age? Gender? Language?

Now I would like to end the interview with some final questions.

5. Firstly, is there anything you want to say? Is there anything we haven't asked about that you think we should talk about?
6. Should I have asked something more/something else?
    a. How do you think this interview went?
7. Could we have done anything better?
    a. How did you feel about our behavior?
8. Do you have any questions for us?

If we have time to spare.

We have some time to spare. Would you be interested in seeing a demo with the robot?

- Can you tell us what you thought of the demo?
- Can you think of any other ways this robot could be used to help you or people you know to be more healthy?

Check the FINDRISC-Score

Finally, I just want to double-check with you that the robot really understood what you said. The robot understood that you said the following:

- Age
- Height
- Weight

- Waist size

If not: recalculate FINDRISC results.
