# Supplementary material for: User Experiences With a Social Robot for Cardiometabolic Risk Assessment in a Community Setting in Uppsala, Sweden: Qualitative Semistructured Interview Study
Source: J Med Internet Res. 2026 Oct 6;28:e87025. doi: 10.2196/87025 (PMC13641321; doi:10.2196/87025)
Supplement: Supplementary file 2 [file jmir-v28-e87025-s002.pdf]

Multimedia Appendix 2 - Codebook

Theme	Sub-theme	Code	Definition
Self-versus-others: a matter of perception	Age matters – but only if you think it does	Age differences	Discussion of whether participants perceived the usage of the robot to be easier or more difficult depending on their own age.
		Expected or recommended age for usage	Discussion of what age participants would recommend the usage of the robot for screening to and what age they think people would show interest in using the robot and why.
		Expected ability based on users age	Discussion of what users expect of the social robot for the risk assessment based on their age.
	All would use the robot, including women	Expected ability based on users' gender	Discussion of what users expect of the social robot for the risk assessment based on their own gender.
		No difference in gender preferences	Discussion about why participants believe man and women would equally enjoy and use the robot for T2D risk assessment.
		Male perception of female preferences for screening	Discussion about what male participants think about female users´ preferences. If they would use the screening with the robot or not and for what reasons.
		Gender differences	Discussion of whether participants found it easier or more difficult to interact with the robot based on their own gender.
	Excitement, mistrust and ridicule	Trustworthiness of robot	Discussion about whether the participants trust the robot or not.
		Perception of others towards people using the robot	Discussion of what participants believe other people will think about them or others using the robot for health screening.
		Others attitude towards the robot	Discussion about what participants think other community members would think about the robot screening.
		Users attitude towards the robot	Discussion about the emotions the participants felt during the interaction with the robot and how this influenced their attitude towards the robot.
		Recommending the screening to others	Discussion about whether users would recommend the screening to others or not and their reasons to do so.
		Influence of experience with technology on social influence	Discussion about how the level of experience with technology use influences participants attitude towards using the social robot for T2D screening.

	Foreseeing barriers: Immigrant identity and technology use	Differences based on country of origin	Discussion about whether participants believe that foreigners, like themselves, or people born in Sweden would be more likely to use the social robot for screening if it was installed in the community.
		Language skills of others	Discussion about whether participants believe others would use the robot based on their Swedish skills and if they would recommend it to people who do not talk Swedish so well.
		Influence of own age on learning Swedish	Discussion about how age might influence the ease of learning a new language (Swedish) and based on that how age might have influenced participants interaction with the robot in Swedish.
		Provision of explanation and information	Discussion about whether participants believe that people in the community would use the robot for screening based on the amount and content of information given to them or if they would need more explanations and information about the robot screening.
The robot is good, but humans are better.	I need someone like me	User friendliness	Discussion about whether participants believe that the robot is easy to operate or not.
		Wording	Discussion about whether participants perceived the questions and the sentences to be formulated in a manner that is clear to the point and easily understandable or not.
		Uncertainties in interaction	Discussion about any uncertainties users faced during their interaction with the robot.
		Hearing impairment as a barrier for understanding	Discussion about how the hearing impairment of one of the participants influenced his ease of understanding and interacting with the robot for the T2D risk assessment.
		Advantages of conducting screening with human instead of robot	Discussion about what the participants perceive to be the advantages of conducting the T2D risk assessment with a human instead of a robot.
		Similarities between human and robot screening	Discussion about the similarities that the users perceived between conducting the T2D risk assessment with a human and a robot.

		Advantages of conducting screening with robot instead of human	Discussion about what the participants perceive to be the advantages of conducting the T2D risk assessment with a robot instead of a human.
		Amount of questions	Discussion about whether the interaction with the robot met the participants expectations in regard to the amount of questions asked and if they believe that the amount of questions asked is sufficient to determine their T2D risk status.
	Feeling comfortable and safe during the interaction	Personal integrity	Discussion about how the participants perceived the interaction with the robot to promote or hinders personal integrity during the screening process compared to a human.
		Opportunity to communicate in Swedish	Discussion about whether participants see communication with the robot in Swedish as a chance to enhance their Swedish skills as opposed to practising speaking with a human.
		Comparing robot to other technology	Discussion about how users perceive the usage of the robot to be different or similar to other technologies and how this influenced their trust in the robot.
		Appearance of robot	Discussion about whether participants liked the current appearance of the robot (titan face and male voice) or if they would have changed anything, as well as how the current appearance influenced their interaction with the robot.
		Attitude and tone of robot	Discussion about how participants perceived the robot to behave in regards to its tone of voice and way to ask questions and how this made them feel.
		Perceived health benefits of screening	Discussion about whether the participants believe that the screening with the robot brings them benefits for their health.
		Perceived benefits of robot screening in community	Discussion about what participants perceive to be the benefits of using a social robot for T2D screening that is installed in the community in contrast to having to go to a healthcare facility for screening.
	Speaking the same language is easier	Swedish as a language barrier	Discussion of how the Swedish language is or is not a barrier for the interaction with the robot.
		Relationship code: Language and privacy during usage	Discussion about how participants thought the privacy during interaction would be hampered or improved if there would be

			the option to choose different languages vs. if there would not be the option.
		Pace of robot	Discussion about how participants perceived the pace of the asked questions of the robot to be too slow, just right, or too fast.
		Articulation of robot	Discussion about participants perception on the articulation of the social robot and how easy it was to understand it.
		Clarifying option	Discussion about what participants thought about the function of asking the robot to repeat the questions in case they did not understand it the first time.
		Language recognition ability of robot	Discussion about whether the robot was or was not able to understand what the participants were saying.
	Suggested improvements	Additonal technical features to improve understanding	Discussion about what features the participants wished for that could improve the ease of understanding.
		Presentation of results	Discussion about whether participants found the presentation of the risk score to be easily understandable or if they wished for another way of presenting them and interpreting them for them.
		Subsequental tests and advice	Discussion about whether the participants belived that the FINDRISC questionnaire is enough or if they would have wished for the robot to have the ability to provide additional tests and advice regarding T2D.
		Other language options	Discussion about whether participants believed that the robot should have different languages programmed that users can choose from and if yes what kind of languages that would be.
		Privacy during usage	Discussion of what participants believed to be facilitating conditions for ensuring the privacy during the screening.
		Agency in customization	Discussion about if the participants want to choose the appearance of the robot before the start or not.
