# Supplementary material for: User Experiences With a Social Robot for Cardiometabolic Risk Assessment in a Community Setting in Uppsala, Sweden: Qualitative Semistructured Interview Study
Source: J Med Internet Res. 2026 Oct 6;28:e87025. doi: 10.2196/87025 (PMC13641321; doi:10.2196/87025)
Supplement: Supplementary file 3 [file jmir-v28-e87025-s003.pdf]

Multimedia Appendix 4 – Disclosure of prompts and responses of generative AI

Disclosure of the original prompts and responses of generative AI used in the rewriting and editing part of the manuscript:

AI used: Claude Sonnet 4.6

Conversation 14th of June 2026:

Can you translate this entire conversation in English?

21:35

Claude: That's quite a long conversation to translate! Just to make sure I give you what you need — do you want the full translation including all the technical troubleshooting about Zotero and Word, or mainly the parts about the reference list formatting and citations?

You: Everything please

21:35

Claude: Here is the full translation of our conversation:

You: I want to update a Word document in which I have inserted sources using Zotero and created a bibliography at the end. However, I keep getting the message that "Word could not communicate with Zotero." I recently did an update for Zotero and changed my Word license from a university license to a family license. Is one of these the cause? What can I do to fix the error?

Claude: This is a classic problem that can be caused by both changes. Let me break it down:

Likely causes

1. Word license change (most likely cause)
When you switch from a university license to a family license, Word is often reinstalled or reset. Add-in settings are lost in the process – the Zotero Word add-in is still present in Zotero, but is no longer correctly registered in Word.

2. Zotero update
Some Zotero updates require a reinstallation of the Word add-in, especially with major version changes.

Solution: Reinstall the Zotero Word add-in

This is the solution in almost all cases and is quick:

1. Open Zotero (without Word)
2. Go to Edit → Preferences → Cite → Word Processors
3. Click "Install Microsoft Word Add-in"
4. Then restart Word
5. A Zotero tab should now appear again in the Word menu bar

If that doesn't help

Check the add-in manually in Word:

- Open Word → File → Options → Add-ins
- At the bottom under "Manage" → COM Add-ins → Go
- Check if "Zotero Word for Windows Integration" is listed and enabled

Is Zotero running in the background?
Word communicates with Zotero through a local port – so Zotero must be open when you insert sources or update the bibliography in Word.

Firewall/Antivirus:
Sometimes these block local communication between Word and Zotero. Try disabling them briefly to rule this out.

Your bibliography is preserved
Don't worry – the Zotero fields already inserted in your document will not be lost through these steps. The connection is simply being re-established.

Try the add-in reinstallation first – this solves the problem in 90% of cases!

You: Thank you, it works now. However, it no longer updates the order of the sources. At least not the ones I inserted before – when I insert a new source now it labels it as 1 and not as the number before it. What can I do to fix this without having to re-link all sources? Or do I have to?

Claude: This sounds like a corrupted field data problem – the old Zotero fields are still in the document, but Zotero no longer recognizes them as connected to the new Zotero context.

What likely happened

By reinstalling the add-in, Zotero internally started a new "session." The old citation fields are still there, but Zotero treats them and the new fields as separate groups.

Solution: Update document data (without re-linking)

Step 1: Re-link Zotero fields
In Word in the Zotero tab:

1. Click "Edit Bibliography"
2. Then click "Refresh" in the Zotero tab

If that doesn't help:

Step 2: Reset Document Preferences

1. In the Zotero tab → "Document Preferences"
2. Switch the citation style once (e.g. to another one), confirm
3. Then switch back to your original format
4. Zotero has to reprocess all fields and should correct the order

If that still doesn't help

Unlink & Relink via RTF:

1. Save the document as .rtf
2. Open the RTF file again in Word
3. In Zotero → "Unlink Citations" – WARNING: This turns Zotero fields into plain text, so save a copy first!

This is the last resort, as you can no longer edit the sources through Zotero afterwards.

Most important first step

First just try the "Refresh" button in the Zotero tab in Word – this often fixes ordering problems immediately, as Zotero then goes through the entire document again and rebuilds the numbering from scratch.

You: The first two options don't work. Unfortunately I don't understand the last suggestion – how exactly is it supposed to help me if I then can't change all the sources through Zotero anymore? Do I have to do everything manually?

Claude: You are absolutely right, the last suggestion was not helpful – sorry for the confusion! That would only make the problem worse.

A few more things you can try first

1. Update all fields in the document (Word-internal)

- Select everything with Ctrl + A
- Then press F9 – this forces Word to recalculate all fields
- Then click "Refresh" in Zotero again

2. Copy document into a new document

- Select everything (Ctrl + A) and paste into a new empty Word document
- Zotero sometimes recognizes the fields better in a fresh document and renumbers correctly

3. Clear Zotero cache

- Close Zotero
- Delete the "cache" folder in the Zotero data folder
- Restart Zotero and then press "Refresh" in Word

If nothing works

Unfortunately it may be that the combination of the Word license change and Zotero update has corrupted the old field data so badly that Zotero can no longer repair it. In that case the cleanest solution would be to re-insert the old citations – but only the old ones that are incorrectly numbered. The new ones already work correctly.

How many old sources are there approximately? That would help assess whether it is still realistically manageable manually.

You: There are 52 old sources that are no longer correctly linked.

Claude: 52 sources to re-link manually is really a lot of work – we want to avoid that. Let's try one more approach before giving up:

Last attempt: Via a new document copy

1. Save the current document as a new copy (important as a backup!)
2. Open the copy
3. In the Zotero tab → "Document Preferences"
4. Switch the citation style to any other one (doesn't matter which)
5. Click OK – Zotero will then reprocess all fields in the entire document
6. Immediately after go back into "Document Preferences" and switch back to your original style
7. Click "Refresh"

The difference from before: The double style switch forces Zotero to completely re-read and renumber all 52+ fields – not just the new ones.

If that doesn't work either

Then my honest recommendation would be: Post your problem on the Zotero Forum – it is very active and the developers respond there themselves. A combination of Word license change + Zotero update with corrupted fields is a specific problem for which the developers may know a direct tool or workaround that I am not aware of.

Did the style switch trick change anything?

You: Where do you find the Zotero data folder and where and how can you delete the cache?

Claude: Finding the Zotero data folder

Easiest way through Zotero itself:

1. Open Zotero
2. Edit → Preferences → Advanced
3. There you can see the path under "Data Directory" and can click directly on "Show Data Directory" – this opens the folder directly in Explorer

Default path (if Zotero won't open):
C:\Users\YOURNAME\Zotero

Deleting the cache

Once you are in the data folder:

1. Close Zotero completely first (also check the system tray)
2. Look for the folder named "cache"
3. Delete the entire "cache" folder (or just the contents)

4. Restart Zotero
5. Press the "Refresh" button in the Zotero tab in Word

Important beforehand

Make sure to make a copy of your Word document before pressing Refresh – just to be safe in case something unexpected happens.

Have you found the data folder?

You: [Uploaded screenshot] Thank you, however I only see this folder and no cache folder.

Claude: That is the storage folder, not the main data folder. You are one level too deep!

How to go one level up

The storage folder you see is part of the Zotero data folder. Simply go one step back:

1. In Explorer at the top in the address bar – click on the folder one level up (i.e. the folder that contains "storage")
2. It should then look like this: C:\Users\YOURNAME\Zotero\

There you should then see several folders and files, including:

- 📁 storage ← where you just were
- 📁 cache ← what we are looking for
- 📁 locate
- 📄 zotero.sqlite
- and others

If you don't see a "cache" folder there, that is also fine – it just means Zotero hasn't created a cache at the moment, and this step is unnecessary.

In that case I would directly recommend the style switch trick from my previous message, if you haven't tried that yet!

You: Thank you, unfortunately none of that works. I will now add them all manually again. Is there a way to display the citations in the text in these brackets [] instead of these brackets ()?

Claude: That can be changed very easily through the citation style – the type of bracket is determined by the chosen style.

How to change it

In the Zotero tab in Word → "Document Preferences"
There switch the citation style

Styles that use square brackets [ ]

- Vancouver → [1], [2], [3]

- IEEE → [1], [2], [3]
- Nature → [1], [2], [3]

Styles that use round brackets ( )

- APA → (Author, Year)
- Chicago → (Author Year)
- Harvard → (Author, Year)

If your desired style is not there

1. In Zotero → Edit → Preferences → Cite
2. Click "Manage Styles" or "Get additional styles"
3. There you can search and download from thousands of styles

What format do you need – just square brackets with numbers, or with author name and year?

You: I need this citation style: [link to JMIR Publications] Is there one that can be downloaded directly into Zotero? I have only found one for EndNote which I don't use.

Claude: Good news – JMIR provides an official Zotero style directly on their support page!

How to install the JMIR style in Zotero

1. Go directly to this link: https://www.zotero.org/styles?q=jmir
2. Click on "Journal of Medical Internet Research"
3. A popup opens – click "Install"
4. Zotero installs the style automatically

Then apply in Word

1. In the Zotero tab → "Document Preferences"
2. Search for "Journal of Medical Internet Research" or "JMIR"
3. Select and confirm

What the style does

Citations in the text appear in square brackets in numerical order of appearance, e.g. [1], [2,3] or [2-13] for consecutive sources. The reference list is automatically numbered at the end of the document.

JMIR itself notes that the style is "untested," but it works well in practice in Zotero – and the comment on the page confirms that it is also compatible with Zotero.

You: [Uploaded reference list] Thank you, can you help me check the reference list to see if it is correct and follows the JMIR criteria?

Claude: I have loaded the JMIR guidelines and thoroughly checked your list. Overall the list looks very good! Here are the points I found:

✅ What is correct

- Numerical order with period (1., 2., 3. ...)
- PMID and DOI are present where available
- ISBN for books present
- Access dates for websites present

⚠️ Problems I found

Ref. 1 – Publisher should not be in the reference. Remove "SAGE Publications." Same applies to other references with publisher names:

- Ref. 3 → Remove "Mary Ann Liebert, Inc., publishers"
- Ref. 8 → Remove "Multidisciplinary Digital Publishing Institute"
- Ref. 11 → Remove "Management Information Systems Research Center, University of Minnesota"
- Ref. 19 → Remove "Springer Healthcare"
- Ref. 51 → Remove "SAGE Publications"

Ref. 10 – DOI and ISBN written together without a space → should be separated

Ref. 12 – Author format strange: "Cardiol J, Ther C, Rao G" – looks like journal abbreviations were entered as author names. Please check if the authors are correct.

Ref. 28 – Book chapter: URL looks like a VitalSource eBook link that may not be publicly accessible. Replace with a publicly accessible link if possible.

Ref. 44 – No DOI and no PMID available. If none is available that is acceptable – but please check again if a DOI exists.

You: Thank you, I would love that.

Claude: [Provided all corrected references 1, 3, 8, 10, 11, 12, 19, 28, 44, 51]

You: Thank you, can you do the same for this citation: Lupton D. 'An Optimal Human Being'...

Claude: Here is the citation in the correct format:

Lupton D. 'An Optimal Human Being': The Body and Self in Self-Tracking Cultures. In: Lupton D, editor. The Quantified Self. Cambridge: Polity Press; 2016. p. 64–87. ISBN: 978-1-

5095-0061-1

Note on Ref. 9: Since you are now citing this book chapter, check whether Ref. 9 (the entire book) is still needed separately to avoid duplicates.

You: Thank you, can you do the same for this citation: Lupton D. The diversity of digital technology use...

Claude: Here is the citation in the correct format:

Lupton D. The Diversity of Digital Technology Use. In: Lupton D, editor. Digital Sociology.

London: Routledge; 2015. p. 117–140. ISBN: 978-1-317-69180-8

Note on Ref. 10: Same as before – since you are now citing a specific chapter, check whether Ref. 10 (the entire book "Digital Sociology") is still needed separately to avoid duplicates.

You: Can you reformulate this in a bit easier English: We acknowledge however that our findings reflect initial perceptions rather than longitudinal adoption trajectories, and have qualified our interpretations accordingly.

Claude: Here are a few options depending on how simple you want it:

Option 1 (slightly simpler):
We acknowledge that our findings reflect first impressions rather than long-term usage patterns, and have interpreted our results with this in mind.

Option 2 (even simpler):
We note that our findings show how participants felt at first, not how they might use the robot over time, and we have taken this into account in our interpretations.

Option 3 (most straightforward):
Our findings capture participants' initial reactions rather than whether they would actually use the robot long-term, and we have considered this limitation when interpreting our results.

Which style fits best for your paper?

Conversation 16th of June 2026:

You: Can you help me rewrite this sentence: "The first theme encompasses how social norms and participants’ own assumptions influence their willingness to engage with the robot for health screening, reflecting findings related to the UTAUT outcome of behavioural intention, which were in this theme influenced by the constructs of social influence and performance expectancy." It should be made into 2 sentences since it is too long and confusing like this and I would like the part about "which were in this theme influenced by the constructs of social influence and performance expectancy" written more easily understandable. Can you give me some options?

Claude: Here are a few options:

Option 1 (straightforward split)
"The first theme explores how social norms and participants' own assumptions shape their willingness to engage with the robot for health screening. This reflects the UTAUT outcome

of behavioural intention, which in this theme was driven by the constructs of social influence and performance expectancy."

Option 2 (more natural flow)
"The first theme explores how social norms and participants' own assumptions shape their willingness to engage with the robot for health screening. This aligns with the UTAUT outcome of behavioural intention, with social influence and performance expectancy emerging as the key contributing constructs."

Option 3 (slightly more formal)
"The first theme concerns how social norms and participants' own assumptions influence their willingness to engage with the robot for health screening. Findings in this theme relate to the UTAUT outcome of behavioural intention, shaped here specifically by social influence and performance expectancy."

All three keep your meaning intact — the main differences are in how explicitly they name the UTAUT link and how they phrase the relationship between the constructs and behavioural intention. Option 2 tends to read the most smoothly for academic writing.

You: Thank you, I like the second the best as well, however I feel like it is not clear enough that social influences and performance expectancy are the main drivers for this theme only and not for the whole research. Can you encompass that better?

Claude: Here are a few ways to make that clearer:

Here are a few ways to make that clearer:

Option 1 (adds "within this theme")
"The first theme explores how social norms and participants' own assumptions shape their willingness to engage with the robot for health screening. This aligns with the UTAUT outcome of behavioural intention, with social influence and performance expectancy emerging as the key contributing constructs **within this theme**."

Option 2 (restructures to front-load the theme-specific framing)
"The first theme explores how social norms and participants' own assumptions shape their willingness to engage with the robot for health screening. Within this theme specifically, behavioural intention — as defined by UTAUT — was primarily driven by social influence and performance expectancy."

Option 3 (makes the scope limitation more explicit)
"The first theme explores how social norms and participants' own assumptions shape their willingness to engage with the robot for health screening. This aligns with the UTAUT outcome of behavioural intention, which in the context of this theme was particularly shaped by social influence and performance expectancy."

Option 2 is probably the strongest here — by leading the second sentence with "within this theme specifically," it makes it immediately clear that you are scoping the claim before even naming the constructs.

You: Thank you. Can you help me combine these two sentences: "On one hand, participants reflected on their own willingness to interact with the robot, while on the other, they described how they expected others to react to the robot screening" and "The first theme explores how social norms and participants' own assumptions shape their willingness to engage with the robot for health screening." as they overlap?

20:12

Claude: Here are a few options that merge the two without losing either idea:

Option 1 (clean and concise)
"The first theme explores how participants' own willingness to engage with the robot for health screening and their expectations of others' reactions to it were shaped by social norms and personal assumptions."

Option 2 (preserves the "on one hand / on the other" contrast)
"The first theme explores how social norms and personal assumptions shaped participants' engagement with the robot for health screening — both in terms of their own willingness to interact with it and their expectations of how others would react to it."

Option 3 (more detailed)
"The first theme explores how social norms and personal assumptions influenced participants' relationship with the robot for health screening, capturing both their own willingness to interact with it and their expectations of others' reactions to it."

Option 2 is likely the strongest, as the dash neatly introduces the two-part distinction from your original sentence while keeping the focus of the first sentence intact.

You: Can you help me formulate this more smoothly: "This second theme describes participants’ experience of robot interaction, reflecting findings related to the UTAUT outcome of use behavior. It encompasses participants´ perceived differences between conducting the screening with a human versus a robot and experiences with language barriers during the interaction, both reflecting the constructs of effort expectancy and performance expectancy. Participants' feelings of safety and trust when interacting with the robot emerged inductively, extending beyond the core constructs of UTAUT. Suggested improvements for the future installation of the robot in the community reflect the UTAUT construct of facilitating conditions." I feel like the last two sentences could be better connected to the first half of the text.

20:27

Claude: Here are a few options that better connect all four sentences into a cohesive paragraph:

Option 1 (uses a bridging phrase for the last two sentences)
"This second theme describes participants' experience of robot interaction, reflecting findings related to the UTAUT outcome of use behavior. It encompasses participants' perceived differences between conducting the screening with a human versus a robot and experiences with language barriers during the interaction, both reflecting the constructs of effort expectancy and performance expectancy. Beyond these core constructs, participants' feelings of safety and trust when interacting with the robot emerged inductively, extending the theme further. Participants also suggested improvements for future installation of the robot in the community, reflecting the UTAUT construct of facilitating conditions."

Option 2 (flows as one coherent arc)
"This second theme describes participants' experience of robot interaction, reflecting findings related to the UTAUT outcome of use behavior. It encompasses participants' perceived differences between conducting the screening with a human versus a robot and experiences with language barriers, both tied to the constructs of effort expectancy and performance expectancy. The theme further extends beyond these core UTAUT constructs, incorporating participants' feelings of safety and trust — which emerged inductively — as well as their suggestions for future improvements to the robot's community installation, the latter reflecting the UTAUT construct of facilitating conditions."

Option 3 (separates UTAUT-aligned and inductive findings more explicitly)
"This second theme describes participants' experience of robot interaction, reflecting findings related to the UTAUT outcome of use behavior. Within the UTAUT framework, it encompasses participants' perceived differences between conducting the screening with a human versus a robot and experiences with language barriers, reflecting the constructs of effort expectancy and performance expectancy, as well as their suggestions for future improvements to the robot's community installation, reflecting facilitating conditions. Additionally, participants' feelings of safety and trust when interacting with the robot emerged inductively, extending the theme beyond the core UTAUT constructs."

Option 2 is likely the strongest for flow, as it builds naturally from the UTAUT-grounded findings toward the inductive ones, ending on facilitating conditions as a forward-looking note. Option 3 works well if you want to draw a clear structural line between what was UTAUT-derived and what was inductive.
